# Synthesis and Characterization of Chitosan Particles Loaded with Antioxidants Extracted from Chia (*Salvia hispanica L*.) Seeds

**DOI:** 10.1155/2021/5540543

**Published:** 2021-06-14

**Authors:** Gema Morales-Olán, Silvia Luna-Suárez, Juan De Dios Figueroa-Cárdenas, Monica Corea, Marlon Rojas-López

**Affiliations:** ^1^Instituto Politécnico Nacional, Centro de Investigación en Biotecnología Aplicada, Ex Hacienda De San Juan Molino, Carretera Estatal Santa Ines, Tecuexcomac-Tepetitla. Km. 1.5, Tepetitla, Tlaxcala 90700, Mexico; ^2^CINVESTAV Unidad Querétaro, Libramiento Norponiente No. 2000, Fracc Real de Juriquilla, Querétaro, Qro 76230, Mexico; ^3^Instituto Politécnico Nacional, Escuela Superior de Ingenieria Química e Industrias Extractivas, UPALM, Zacatenco, Ciudad de México 07732, Mexico

## Abstract

Chia (*Salvia hispanica* L.) seeds contain antioxidants with great benefits for health and are widely used in the food industry. Antioxidants can be degraded by environmental factors, decreasing their biological activity. Their encapsulation in chitosan (CH) particles represents an alternative to protect them and increases their application. The encapsulation efficiency (%EE) of the antioxidants in the CH particles depends on the synthesis conditions. In this study, two methods for encapsulation of chia extract in chitosan particles were evaluated: method A, 0.05% CH in 1% acetic acid was mixed with 0.07% of tripolyphosphate (TPP) and method B, 0.3% CH in 2% acetic acid was mixed with 1% TPP. The results showed that the %EE decreased with the concentration of the extract, and the FTIR analysis suggested that the compounds of the extract were adsorbed on the surface of the particles. Dynamic light scattering and zeta potential analysis showed that the particles of method A are unstable and with a tendency to agglomerate, and the particles of method B are stable. The highest %EE was obtained with 0.2 mg·mL^−1^ (method A) and 1.0 mg·mL^−1^ (method B) of the extract. The higher loading capacity (%LC) (16–72%) was exhibited by the particles of method A. The best particle yield (62–69%) was observed for method B. The particles with the extract adsorbed showed antioxidant activity (5–60%) at 25°C; however, in the particles with the extract encapsulated, the activity increased after subjecting to acidic conditions at 40°C due to the breakdown of the particles. The results obtained will allow choosing the appropriate conditions for the synthesis of chitosan particles loaded with chia extracts with specific characteristics (%EE, %LC, size, and type) according to their future applications. The particles could be used in food and pharmaceutical industries and even in edible films for food packaging.

## 1. Introduction

Chia (*Salvia hispanica* L.) seeds present great benefits for nutrition and human health. These seeds provide proteins, carbohydrates, fatty acids, and fiber [1], and they are an important source of phenolic compounds, such as kaempferol (0.403 mg·g^−1^ of seed), quercetin (0.248 mg·g^−1^ of seed), chlorogenic acid (0.102 mg·g^−1^ of seed), myricetin (0.010 mg·g^−1^ of seed), and gallic acid (0.0116 mg·g^−1^ of seed) [2–4]. It has been described that phenolic compounds protect humans against several chronic degenerative diseases [5] and antioxidants are also used as additives for food preservation. However, an important disadvantage is their instability during the processing, distribution, storage, and consumption of food [6]. They can be degraded by various factors (temperature, light, pH, enzymes, and other nutrients) [7], limiting their activity and potential health benefits. To avoid these phenomena, encapsulation is used, which allows trapping active substances within a material, improving its bioavailability, and facilitating the application of the plant extracts [8]. Protected by the particles, the phenolic compounds can be used in manufactured nutraceutical functional foods in the pharmaceutical and cosmetic industry. Among the polymeric materials applied for encapsulation, the CH composed of *β*-(1–4)-linked D-glucosamine (deacetylated unit) and N-acetyl-D-glucosamine (acetylated unit) presents ideal characteristics and is biocompatible, biodegradable, nontoxic, and inexpensive [9]. The synthesis of chitosan particles can occur through many routes, but the ionic gelation reaction is one of the most widely used. The encapsulation of extracts and essential oils from plants in chitosan nanoparticles has been reported [9, 10]. In the literature, there are numerous reports on the encapsulation of chia seed oil using techniques such as layer-by-layer electrostatic deposition of chitosan [11] and atomization and lyophilization using soy protein gum arabic and maltodextrin [12, 13]. In addition, the use of chia seed mucilage as a wall material to encapsulate the oils extracted from the same seed has been evaluated [14, 15]. However, there is no information about the loading of hydroalcoholic antioxidant chia extract into chitosan nanoparticles. Encapsulation of a specific compound of the extract is desirable, but separative methods make the process more expensive and, in some cases, low extraction yield is obtained. Furthermore, it has been reported that the combination of different compounds in an extract can generate a synergy, that is, an increase in the biological activity because they act together [16]. The %EE of the extracts of chitosan particles synthesized by ionic gelation depends on the molecular weight of the polymer, the degree of deacetylation, the concentration of precursors, the pH of the solution, the relationship between the precursors, as well as the affinity between the extract and the CH [17, 18]. Therefore, it is necessary to evaluate different conditions to find efficient encapsulation methodologies. The aim of this work was to evaluate two methods of encapsulation of the extract of chia seeds in chitosan particles synthesized under the ionic gelation methodology. The %EE, %LC, and the particle yield (%PY) of the particles were determined using different concentrations of the extract (0.2–15 mg·mL^−1^). The particles were also characterized by FTIR, dynamic light scattering, zeta potential, and morphologically by SEM. In addition, the effect of temperature (25°C and 40°C) and pH (6 and 10) on particles and the antioxidant capacity was evaluated.

## 2. Materials and Methods

### 2.1. Materials

Medium-molecular-weight CH (190–310 kDa) with a degree of deacetylation of 75–85%, TPP, glacial acetic acid, 2,2-diphenyl-1-picrylhydrazyl (DPPH), and ethanol were obtained from Sigma Aldrich (Sigma Co., San Luis, E.U.). Chia seeds were purchased from a local market in Puebla City, Mexico. They were cleaned manually and were passed through a 20-mesh screen. The flour was generated by grinding the seeds in a food processor and passing them through a 35-mesh screen. The flour was stored at 4°C until its use.

### 2.2. Obtaining of Chia Seeds Extract

To obtain the chia extract, 0.5 g of chia flour was mixed with 3 mL of the 80% aqueous ethanol solution for 24 h at 25°C and centrifuged at 6,000 rpm for 10 min. The alcohol of the extract was removed by using Rotavapor and the water by lyophilization. The extract was stored at 4°C.

### 2.3. Synthesis of Chitosan Particles and Chia Extract-Loaded Particles

The particles were prepared by ionic gelation of CH with TPP. The methods described by Antoniou et al. [19] (method A) and Nadirah et al. [20] (method B) with some modifications were used to evaluate the encapsulation of chia extract in chitosan particles. In method A, 0.05% (w/v) CH was dissolved in 1% (v/v) aqueous glacial acetic acid with pH 4.8. 10.0 mL of the aqueous solution of TPP (0.07% w/v) was added dropwise to 42.0 mL of the CH solution under vigorous magnetic stirring (1000 rpm) at room temperature. For the synthesis of the particles that incorporate the chia extract, different concentrations of the extract (0.2, 0.3, 0.4, 0.5, 1.0, 2.0, 3.0, 4.0, 5.0, and 10.0 mg·mL^−1^) were prepared in the TPP solution with agitation until the dissolution is completed. The solution extract-TPP was added dropwise to the CH solution under vigorous magnetic stirring at room temperature for 10 min. In method B, 0.3% (w/v) CH was dissolved in 2% (v/v) of acetic acid. The nanoparticles were obtained upon the addition of 4.8 mL of 1% TPP (w/v) into 47.2 mL of the CH solution (pH 2.8) under vigorous magnetic stirring at room temperature for 1 h. Several concentrations of the extract (1.0, 2.0, 3.0, 4.0, 5.0, 10.0, and 15.0 mg·mL^−1^) in the TPP solution were added to the CH solution to obtain the loaded particles. In both methodologies, the particles were recovered by centrifugation at 13,000 rpm for 30 min at 25°C. Three washes with deionized water were performed to remove the remnants of the synthesis. The particles were lyophilized and kept at 4°C until further analysis. The particles without extract were named as blank particles.

### 2.4. Encapsulation Efficiency (%EE), Nonencapsulated Extract (%NE), Loading Capacity (%LC), and Particle Yield (%PY) of the Particles

The %EE was determined by analyzing the supernatant of the chitosan particles by UV-Visible spectrophotometry at a wavelength of 320 nm. This is the wavelength of maximum absorption of the chia extract that was obtained by a UV-Visible scan (200–900 nm). In this wavelength, the precursors CH and TPP did not show absorbance. A calibration curve was generated with the extract (0–1.5 mg·mL^−1^, *y* = 1.408*x* + 0.0055, *R*^2^ = 0.99) to calculate the concentration of the extract in the supernatant. The %EE, %LC, and %PY of the particles were calculated using equations (1)–(3), respectively. The %NE was obtained by difference with %EE.

(1) $$\begin{array}{c} \%\mathrm{EE=}\left(\frac{\text{weight of loaded chia extract}}{\mathrm{weight}\;\mathrm{of initial chia extract}}\right)x100\;, \end{array}$$

(2) $$\begin{array}{c} \%\mathrm{LC=}\left(\frac{\text{weight of loaded chia extract}}{\text{weight of sample}}\right)x100, \end{array}$$

(3) $$\begin{array}{c} \%\mathrm{PY=}\left(\frac{\text{nanoparticles weight}}{\text{total solids weight }\left(\text{CH}+\text{TPP}+\text{chia extract}\right)}\right)x100. \end{array}$$

### 2.5. Structural Characterization of the Particles by FTIR

The lyophilized particles were characterized using an FTIR spectrometer (Bruker Vertex 70, Germany) equipped with an attenuated total reflectance (ATR) accessory. The spectral measurements were recorded in the wavenumber range between 4000 and 500 cm^−1^.

### 2.6. Particle Morphology and Size

The particles were examined using the scanning electron microscope SEM-FE-JOL 7610F (Tokyo, Japan) with an Oxford EBSD detector operating at a 2.0 kV voltage acceleration and a secondary electron detector (SEI). The samples were mounted dry on a carbon tape and coated with Au/Pd. SEM images were analyzed with ImageJ 1.52a program (National Institute of Health, USA) to determine the size of the particles.

### 2.7. Dynamic Light Scattering and Zeta Potential

The distribution particle diameter and zeta potential (*ζ*) of the synthesized particles were measured using a Zetasizer Nano ZSP (Malvern Instruments, United Kingdom). The samples were centrifuged and redispersed in distilled water. The measurements were made in triplicate at 25°C.

### 2.8. DPPH Radical Scavenging Activity of the Particles

The antioxidant capacity was determined by the DPPH radical with the methodology proposed by Parejo et al. [21] with some modifications. The particles were mixed with a 100 mM DPPH methanol solution, placed under stirring for 30 min in the dark. Then, they were centrifuged at 6,000 rpm for 1 min and the supernatant was measured at 517 nm. The percentage of the DPPH scavenging activity was determined using equation (4). The results show the mean of three replicates ± SD.

(4) $$\begin{array}{c} \text{DPPH scavenging activity }\left(\%\right)=\left[\frac{\mathrm{absorbanc}e_{\mathrm{control}}\;-\mathrm{absorbanc}e_{\mathrm{sample}}}{\mathrm{absorbance}{\;}_{\mathrm{control}}}\right]\times 100. \end{array}$$

### 2.9. Effect of Temperature and pH on the Particles and the Antioxidant Capacity

The effect of the temperature was evaluated by placing the particles in an oven at 40°C and room temperature (25°C) for 24 h. The treatment at acidic and basic pH consisted of mixing 1.0 mL of the acidic (pH 6) and basic (pH 10) solutions, respectively, with the particles (1.0 mg). The samples were vortexed for 1 min. The mixture was placed in an oven at 40°C for 24 h. The percentage of the DPPH scavenging activity was determined with the methodology described above.

### 2.10. Statistical Analysis

Results were expressed as mean ± standard deviation (SD) and were compared using the analysis of variance (ANOVA) and the Tukey test. Values of *p* < 0.05 were indicative of significant differences.

## 3. Results and Discussion

### 3.1. Encapsulation Efficiency (%EE)

**Figure 1:**
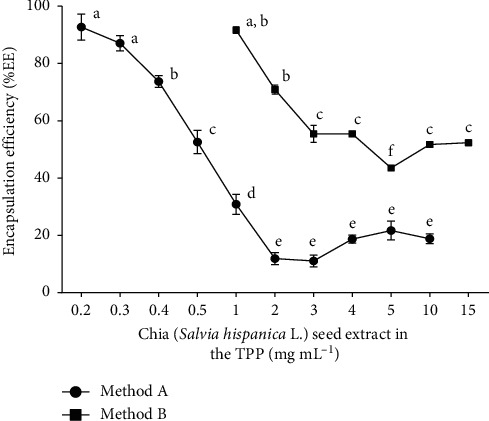
Encapsulation efficiency (%EE) curve of the chia (*Salvia hispanica* L.) seeds extract in chitosan particles synthesized with the methods A and B. Means with different letters are significantly different (*p* < 0.05) according to the Tukey test.

Figure 1 shows the curve of the %EE of the chia extract in the chitosan particles synthesized by methods A and B by dissolving different concentrations of the extract in the TPP. The extract of chia was obtained with 80% aqueous ethanol because in previous studies it was determined that in these conditions, the extract has a higher content of phenolic compounds, DPPH radical scavenging activity, and good solubility in water [22].

In the curve, a similar dependence between the %EE and the concentration of the chia extract was obtained for both methodologies. The %EE decreased with the increasing concentration of the extract. Comparable results were reported by Luo et al. [23], Nallamuthu et al. [18], and Wu et al. [24]. Luo et al. [23] explain that when the concentration of the active substances increases, it is only electrostatically adsorbed on the surface of the chitosan particles and the extract can separate easily during centrifugation, so the %EE decreases. The highest %EE was quantified with a concentration of 0.2 mg·mL^−1^ with method A. The %EE begins to decrease with a concentration of 0.4 mg·mL^−1^ of extract, and with 2.0 to 10.0 mg·mL^−1^, %EE is maintained at about 19%. On the other hand, with method B, the highest %EE was presented with a concentration of 1.0 mg·mL^−1^ of the extract; therefore, lower concentrations of the extract with this method were not considered. The %EE began to decrease with a concentration of 2.0 mg·mL^−1^, and from this, no significant differences were found in the %EE with the concentrations of extract from 3.0 to 15.0 mg·mL^−1^. The %EE obtained with the extract concentrations of 0.2 to 0.4 mg·mL^−1^ (method A) and 1.0 to 2.0 mg·mL^−1^ (method B) were higher than that reported in the encapsulation of other compounds such as chlorogenic acid [18] and ferulic acid [25] in chitosan particles. The statistical comparisons between the %EE values obtained with methods A and B (Figure 1) showed that there are no significant differences between the %EE of the particles synthesized with 0.2 and 0.3 (method A) and 1.0 mg·mL^−1^ (method B), between the particles with 0.4 (method A) and 2.0 mg·mL^−1^ (method B), as well as among the particles synthesized with 0.5 (method A) and 3.0, 4.0, 10.0, and 15.0 mg·mL^−1^ (method B) of the chia extract. However, the main differences found between the particles are the amounts of encapsulated extract. These differences depend directly on the conditions of synthesis. As detailed in the Materials and Methods section, for the evaluated methods A and B, CH with the same molecular weight and degree of deacetylation was used. The mass ratio between the precursors (CH : TPP, 3 : 1) and the temperature of the synthesis were the same, but the concentration of the precursors (CH and TPP), the pH of the CH solution, and the stirring time were different. Method B uses seven times more amount of CH and TPP. Method B uses a higher concentration of CH, which increases the viscosity of the solution. It has been reported that an increase in viscosity hinders the mobility of the molecule to be encapsulated around the CH chain, obtaining low concentrations of the extract in the particles.

The effect of pH on the encapsulation of the extracts in chitosan particles has not been studied. However, in the encapsulation of some polar molecules, it is mentioned that the increase in the pH of the CH solution causes that the molecules gain a negative surface charge. This negative charge favors the electrostatic interaction with the CH (positive charge); therefore, a higher %EE is obtained [26]. The pH of the CH solution in method A (pH 4.8) is higher than the pH of the solution of method B (pH 2.8), which may favor the encapsulation of a greater amount of extract. Regarding the stirring time during the synthesis of the particles, Chopra et al. [27] reported a decrease in the amount of streptomycin encapsulated in chitosan particles by increasing the agitation time of the synthesis. In method B, the magnetic stirring time is longer than in method A. A longer stirring time could cause separation of the extract that is weakly bound to the nanoparticles.

### 3.2. Nonencapsulated Extract (%NE), Loading Capacity (%LC), and Particle Yield (%PY)

The %NE, %LC, and %PY were determined in the particles synthesized with concentrations of the extract of 0.2, 2.0, and 10.0 mg·mL^−1^ (method A) and 1.0, 4.0, and 15.0 mg·mL^−1^ (method B) (Table 1). These samples were chosen because they generated significant changes in the %EE curve and correspond to the low, medium, and high concentrations of the chia extract (Figure 1). %LC is an important parameter in the particles that encapsulate active substances because it indicates the percentage of extract in the dry particles; thus, the dose of the particles depends on this parameter [18]. In both methodologies, it was observed that as the amount of extract increases, the %LC also increases. Woranuch and Yoksan [28] obtained similar results in the encapsulation of eugenol in chitosan nanoparticles.

The chia extract is more retained in the chitosan particles made under the conditions of synthesis of method A. The %LC of the particles synthesized with method B was lower than that with method A. The particles synthesized with 10.0 mg·mL^−1^ of chia extract with method A presented the highest %LC. However, the %EE is low, 18.8%, so during the synthesis of the particles, 81.2% of the extract added is lost, as shown in %NE. This represents a disadvantage in its production. On the other hand, for the particles prepared with a concentration of 0.2 mg·mL^−1^ of extract, the %LC is 16.2% and, in these particles, the amount of extract that is wasted is lower. The %LC of the particles synthesized with method A was higher than that reported in the encapsulation of resveratrol and eugenol in chitosan nanoparticles [24, 28]. In method B, the particles that showed the highest %EE managed to immobilize only 3.2% of the extract with a %NE of 8.3%. The particles elaborated with a concentration of 15.0 mg·mL^−1^ presented a higher %LC than the other particles synthesized with the same method, but the loss of extract during the synthesis is 47.6%. As explained previously, the increase in the concentration of CH and TPP, the pH of the CH solution, and the stirring time in method B could limit the interaction between the extract and the precursors, thus generating a low %LC. The particles with the highest %EE elaborated with method A presented a %PY of 47.2%, and no significant differences were found between the yield of the blank particles and those that encapsulate the chia extract. Different results were obtained with method B. The yields of these particles were higher than the yields of the particles made with method A. It was observed that when increasing the concentration of the extract, the yields of the particles decrease lightly in method B. Dudhani and Kosaraju [29] reported that the decrease in particle yields may be caused by the greater competition between the OH groups of phenolic compounds and the phosphate groups of the TPP for their binding with CH amino groups, resulting in not many particles forming.

### 3.3. Structural Characterization of the Particles by FTIR

**Figure 2:**
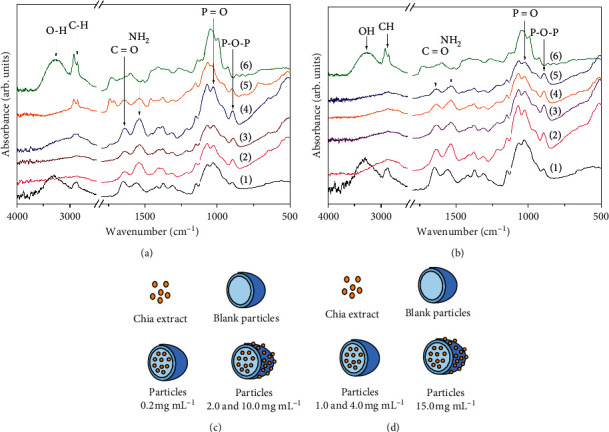
FTIR spectra of chitosan (1), chia extract (6), blank particles (2), and loaded particles with the chia extract synthesized with method A (a): 0.2 (3), 2.0 (4), and 10.0 mg·mL^−1^ (5). Method B (b): 1.0 (3), 4.0 (4), and 15.0 mg·mL^−1^ (5). Schematic representation of the structure of the loaded particles synthesized with the methods A (c) and B (d).

The FTIR spectra of the extract of chia, blank particles, loaded particles, and the precursors of their synthesis (CH and TPP) are shown in Figures 2(a) (method A) and 2(b) (method B). Previously, in aqueous alcohol extracts of chia seeds have been identified compounds such as myricetin, quercetin, kaempferol, and phenolic acids [2, 3]. Characteristic bands of this type of compounds were observed in the FTIR spectrum of the chia extract (Figures 2(a), and 2(b) (6)). The broadband in the region of 3300–3000 cm^−1^ corresponds to the stretching vibrations of the O-H bond. Additionally, the signals located at 2922 cm^−1^ and 2852 cm^−1^, which are associated with the stretching vibrations of the C-H bonds [30] and the bands in the region of 1600–1400 cm^−1^, are attributed to the carbonyl and aromatics groups, basic constituents in the structure of phenolic compounds [31].

The signals in the region of 3700–2800 cm^−1^ in the CH spectra (Figures 2(a), and 2(b) (1)) correspond to the stretching vibrations of the O-H, N-H, and C-H bonds [9, 32]. The bands located at 1648 cm^−1^ and 1557 cm^−1^ arise from the symmetric stretching vibrations of the carbonyl and amino groups, respectively [9, 33].

These signals in the spectra of the particles prepared with methods A (Figure 2(a) (2, 3, 4, 5)) and B (Figure 2(a) (2, 3, 4, 5)), blank and with extract, shift to 1636 cm^−1^ and 1542 cm^−1^, respectively. In addition to that, the signal of the amino group increases in intensity. Furthermore, the signals located at 1024 cm^−1^ and 891 cm^−1^ correspond to the stretching vibration of the phosphate group by the incorporation of TPP. It has been described that these changes are due to the interaction between the amino group of chitosan and TPP, proving the formation of the particles [19, 32, 34]. No differences were found between the spectra of the particles without extract and the particles loaded with 0.2 mg·mL^−1^ of the chia extract synthesized with method A, which suggests that the added extract is encapsulated inside the particle. These results agree with those reported by Zhang et al. [34] in quercetin-loaded chitosan nanoparticles.

In the spectra of the particles synthesized with 2.0 mg·mL^−1^ and 10.0 mg·mL^−1^, two absorption bands at 2922 cm^−1^ and 2852 cm^−1^ were observed (Figure 2(a) (4, 5)). These bands do not appear in the spectra of the blank particles. The bands correspond to the stretching vibration of the C-H bond identified also in the chia extract spectrum. Woranuch and Yoksan [28] explain that the intensity of the signals of the C-H bond increases with the concentration of phenolic compounds in the particles. These results suggest that the extract is not only inside the particles but can be adsorbed on the surface of them. Figure 2(a) also shows the schematic representation of the possible location of the chia extract in the particles synthesized with method A.

No differences were found between the spectra of the particles synthesized with 1.0 mg·mL^−1^ and 4.0 mg·mL^−1^ and the blank ones elaborated with method B (Figure 2(b) (3, 4)). Therefore, there is a total incorporation of the extract within the structure. However, in the spectrum of the particles synthesized with 15.0 mg·mL^−1^, weak signals appear at 2922 cm^−1^ and 2852 cm^−1^ (Figure 2(b) (5)), which suggests the extract could be inside and adsorbed on the surface of the particles. Figure 2(b) also shows the schematic representation of the possible location of the chia extract in the particles synthesized with method B.

### 3.4. Particle Size and Morphology

**Figure 3:**
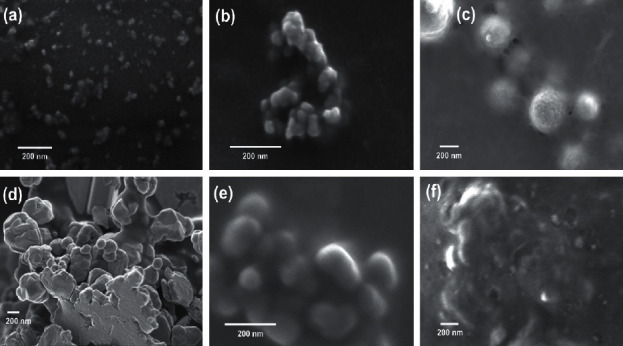
SEM micrographs. Method A: blank particles (a), 0.2 mg·mL^−1^ (b), and 10.0 mg·mL^−1^ (c) chia extract-loaded particles. Method B: blank particles (d), 1.0 mg·mL^−1^ (e), and 15.0 mg·mL^−1^ (f) chia extract-loaded particles.

Figure 3 shows the SEM micrographs of the blank and loaded chitosan particles with chia seeds extract. The size of the particles also depends on various factors such as the relationship between the cross-linker (TPP) and the polymer (CH), the molecular weight of the CH, the concentration, the degree of deacetylation, pH, the mechanical energy used during its preparation, and the salinity of the solvents [35].

Method A produces blank particles with an average particle size of 31 ± 9 nm (Figure 3(a)). These particles have smaller sizes than those reported by Antoniou et al. [19]. The differences in particle size may be due to the lower molecular weight (50–100 kDa) of the chitosan used by these authors, compared with that used in this investigation (190–310 kDa). In other reports, where the effect of the molecular weight of CH on particle formation has been evaluated, it has been observed that high molecular weights generate small particles. This phenomenon occurs because, during the magnetic stirring of the chitosan and TPP mixture, the strong flux to which the polymer is subjected can have enough energy to degrade the molecule into small fragments that generate smaller particles [17, 36]. However, although the formation of individual nanoparticles was observed, also is noticeable the formation of agglomerates between them. The particles synthesized with 0.2 mg·mL^−1^, whose %EE is the highest, showed an average size of 39 ± 8 nm (Figure 3(b)). No significant differences were found between the size of the particles without and with 0.2 mg·mL^−1^ of chia extract. The spheric morphology of the nanoparticles was observed, but again, as in the previous case showing agglomerates. The particles obtained with a concentration of the extract of 10.0 mg·mL^−1^ showed an average size of 328 ± 91 nm (Figure 3(c)). This increase in particle size is due to the extract loaded in chitosan particles, as reported by other authors [18, 25]. In this case, is not evident the formation of agglomerates, but large individual particles.

Method B generated blank particles that agglomerate between them during the dry process. An estimation of the average size of these blank particles was complicated, and at the same time, we think this is vague because of their agglomerated nature (293 ± 65 nm) (Figure 3(d)). The estimation size of these particles was similar to that reported by Nadirah et al. [20] (216 nm). These authors used CH with a molecular weight of 100–300 kDa as a precursor. The particles synthesized with 1.0 and 15.0 mg·mL^−1^ of the chia extract, according to the SEM micrographs, presented average sizes of 42 ± 18 nm (Figure 3(e)) and 76 ± 24 nm (Figure 3(f)) respectively, although for this case also it could be vague because the morphology in the image is not very clear. In method A, the increase in the concentration of the extract in the synthesis increased the size of the particles, whereas a contrary behavior was observed in the particles synthesized with method B. The decrease in the size of the particles elaborated with method B may be due to the competition between the phenolic compounds and the phosphate groups of the TPP for their union with the amino groups of CH, as explained above.

The initial pH of the CH solution also significantly affects the size of the particles. Antoniou et al. [19] reported that low pH values in the CH solution produce strong positively charged amino groups, which lead to repulsion in the CH chain, resulting in larger particles. The results of these authors are similar to those obtained in this investigation, except for the particles synthesized with 10.0 mg·mL^−1^ (Method A), the particles made with method B (CH solution, pH 2.8) were larger. Stirring time is another factor that has been related to the increase in the particle size because during this procedure the particles can agglomerate [37].

Regarding the morphology of the particles, in some of them, a spherical morphology was observed; in others, the structure is irregular or not very evident. As many authors have reported, chitosan particles tend to form agglomerates, making it difficult to visualize their shape [9, 38].

### 3.5. Dynamic Light Scattering and Zeta Potential

The zeta potential values of the synthesized particles are presented in Table 2. The results show that particles made by method A have potentials between 21.8 mV and 26.3 mV. That means the formed colloid is unstable and with a certain tendency to agglomerate [39].

**Figure 4:**
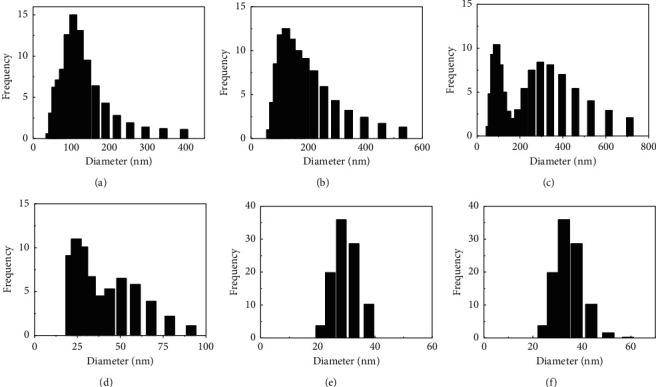
Particle size distributions. Method A: blank particles (a), 0.2 mg·mL^−1^ (b), and 10.0 mg·mL^−1^ (c) chia extract-loaded particles. Method B: blank particles (d), 1.0 mg·mL^−1^ (e), and 15.0 mg·mL^−1^ (f) chia extract-loaded particles.

This also is coincident with the size particle distribution results (Figure 4), where wide distributions are observed in the synthesized systems by Method A. The formation of agglomerates is also corroborated in the SEM micrographs.

The results of zeta potential for synthesized particles with method B revealed colloidal systems with better stability (52.4 ± 1.9 mV and 39.3 ± 0.3 mV) than those synthesized with method A, except for the particles without chia extract (26.0 ± 1.2 mV). This was corroborated with the dynamic light scattering results, where the chia extract loaded particles with concentrations of 1.0 mg·mL^−1^ and 15.0 mg·mL^−1^ have narrow size distributions.

### 3.6. DPPH Radical Scavenging Activity of the Particles

#### 3.6.1. Effect of Temperature

**Figure 5:**
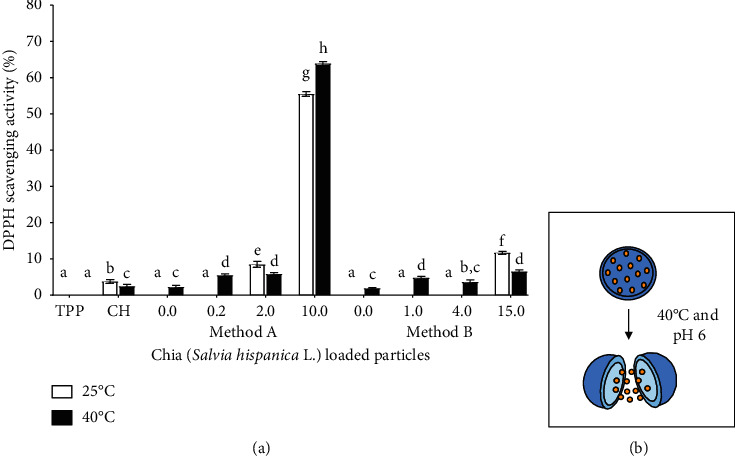
(a) DPPH radical scavenging activity of precursors (CH and TPP), blank particles (0.0 mg·mL^−1^), and chia extract-loaded particles with different concentrations synthesized with the methods A and B after treatment at 25°C and 40°C. The columns with different letters are significantly different according to the Tukey test (*p* < 0.05). (b) Schematic representation of the effect of the temperature and pH on the particles.

The DPPH radical scavenging activities of the precursors, blank particles, and chia extract-loaded particles were evaluated at 25°C and 40°C, as shown in Figure 5. These temperatures were chosen because one of the possible applications of the particles that encapsulate antioxidants is for food preservation, specifically to delay lipid oxidation and the rancid process. These phenomena can occur at temperatures ranging between 25°C and 40°C in some places with extreme temperatures. Furthermore, one of the current applications of the particles with extracts is their incorporation into edible films. In many of the edible packaging manufacturing procedures, the solvent is removed at temperatures of 40°C or more, so it is important to know the response of the nanoparticles at these temperatures.

Between 25°C and 40°C, the TPP showed no activity. However, CH at 25°C presented a DPPH scavenging activity of 3.7% and at 40°C the activity was lower (2.4%). Several authors have described the low antioxidant capacity of CH [40, 41]. The antioxidant activity of CH is due to the protonated amino groups of C2 that react with the antioxidant agent [40]. The particles prepared without extract (blank, 0.0 mg·mL^−1^) in both methods showed no activity at 25°C. The amino group of CH reacts with TPP to form the particle, so linked to the crosslinking agent does not have antioxidant activity. These results agree with those reported by Zhang et al. [34]. The particles synthesized with a concentration of 0.2 mg·mL^−1^ (method A) and 1.0 mg·mL^−1^ and 4.0 mg·mL^−1^ (method B) showed no DPPH scavenging activity at 25°C. The lack of antioxidant capacity of these particles loaded with the chia extract suggests that the phenolic compounds are inside as evidenced by the FTIR spectra (Figure 2). Inside of the particles, the compounds do not interact with the DPPH molecule. In contrast, the particles synthesized with 2.0 and 10.0 (method A) and 15.0 mg·mL^−1^ (method B) of the extract exhibited DPPH scavenging activity at 25°C. The activity of these particles was significantly higher than the blank particles and CH. This agrees with the FTIR spectra of the particles with such concentrations, where spectral features related to the extract were observed, indicating the adsorption of the extract on the surface of the particles (Figure 2). Therefore, the molecules of the periphery can react with the radical, inhibiting it. The highest DPPH radical scavenging activity at 25°C was obtained in the particles synthesized with 10.0 mg·mL^−1^, which also showed a higher %LC. That is, these particles contain a higher percentage of extract. The antioxidant capacity of these particles is higher than that reported by Wu et al. [24] in nanoparticles that incorporate resveratrol.

The particles synthesized with methods A and B showed a greater antioxidant capacity at 40°C than at 25°C. These results suggest that the thermal process at 40°C causes the particle to break (Figure 5(b)). The rupture of the particles without extract leaves free chitosan amino groups that can now interact with the DPPH radical and in the particles loaded with the chia extract causes the release of the extract that can react with the radical. Szymańska and Winnicka [42] mention that one of the environmental factors that affect the stability of chitosan is the temperature. Viljoen et al. [43], in the study on the effect of moisture content, temperature, and time of exposure on the physical stability of CH, found that exposure to 40°C caused a significant loss of moisture, which resulted in a decrease in the hardness and mechanical strength of the particle made with this polymer. Different results were found in the particles synthesized with 2.0 and 15.0 mg·mL^−1^ since a decrease in the antioxidant capacity was observed after treatment at 40°C. The highest DPPH scavenging activity at 40°C was obtained in the particles synthesized with 10.0 mg·mL^−1^ of the extract with method A. Except for the particles synthesized with 10.0 mg·mL^−1^, the DPPH scavenging activity of the particles was less than 10%, and this is because the content of the extract encapsulated in them is small, as demonstrated by the parameter %LC. In the particles synthesized with 0.2 and 2.0 mg·mL^−1^ (method A), approximately 15% is extract, and in those made with 1.0 and 4.0 mg·mL^−1^ (method B), only 3% and 8% are extract. In contrast, the particles made with 10 mg·mL^−1^ contain 5 times more of chia extract (%LC 72.5%) and part of this extract may be adsorbed on the surface. The results show that the release of the chia extract from the chitosan particles is favored at 40°C. Derived from these results, the processes of making edible films by using the casting method can affect the stability of the particles, allowing the extract to be released. The breakdown of these particles could also alter other important properties of films such as their mechanical and barrier properties. This will depend on the characteristics of the polymers used and their interaction with the nanoparticles.

#### 3.6.2. Effect of pH

**Figure 6:**
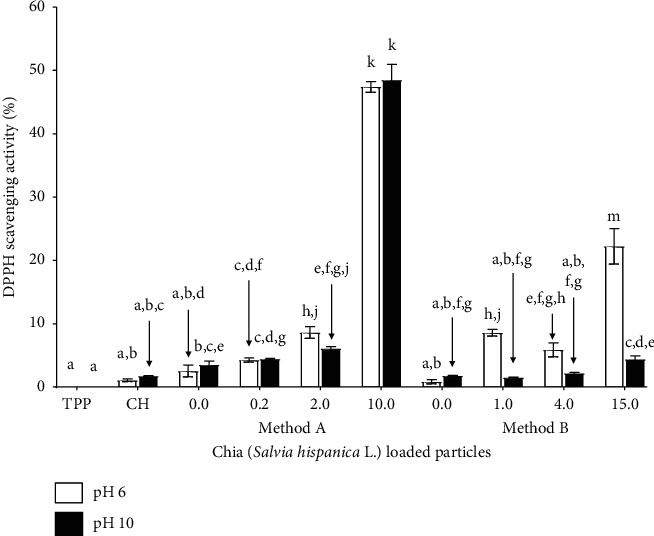
DPPH radical scavenging activity of precursors (CH and TPP), blank particles (0.0 mg·mL^−1^), and chia extract-loaded particles with different concentrations synthesized by methods A and B after treatment with acidic and basic solutions. The columns with different letters are significantly different according to the Tukey test (*p* < 0.05).

Figure 6 shows the DPPH scavenging activity of the precursors and particles with and without extract after treatment with acidic and basic solutions. The particles were subjected to pH 6 because most of the natural and industrialized foods have this acidic pH [44]. As mentioned above, one of the possible applications of the particles is their incorporation into food to prevent oxidation. It was also decided to evaluate pH 10 taking into account another application of the particles, their incorporation in filmogenic solutions for obtaining edible films. In this process, the pH is adjusted to 10 to dissolve the proteins, and under these conditions, the particles are added.

The precursor TPP in acidic and basic conditions did not present DPPH scavenging activity. The antioxidant capacity of CH was lower than that found at 25°C and 40°C. No significant differences were found between the antioxidant activity of CH at pH 6 and 10. The highest DPPH scavenging activity at pH 6 and 10 was obtained in the particles synthesized with 10.0 mg·mL^−1^ extract. Under acidic conditions, the particles made with 2.0 and 10.0 mg·mL^−1^ (method A) and those synthesized with 1.0, 4.0, and 15.0 mg·mL^−1^ (method B) of the extract presented a higher antioxidant activity than the blank particles. Thus, the release of the chia extract can occur in acidic conditions. It has been described that at low pH (<pH 6.3), the amines of chitosan are protonated [45] and the CH of the particles can be solubilized when treated with acidic solutions so that the particle can be destroyed leaving the extract free. Under basic conditions, only significant differences were found between the antioxidant capacity of the particles synthesized with 10.0 (method A) and 15.0 mg·mL^−1^ (method B) of extract and the blank particles. It has been reported that at high pH (>pH 7), CH is insoluble (pKa 6.5) and the amines of this polymer are deprotonated and act as nucleophilic, so the pair of nonshared electrons can react with other molecules present in the medium [45]. This may be the cause of the low antioxidant capacity of the particles treated in a basic medium, mainly in the particles synthesized with method B. These particles present a greater content of CH and lower %LC. The results suggest that the particles synthesized with method A could be used for the preservation of acidic or basic foods since the extract can be released under these conditions. Particles made with method B can be used in acidic environments. On the other hand, in filmogenic solutions for food packaging, the particles could remain embedded in their surface without releasing the extract. This extract could later be released under certain environmental conditions or by contact with food.

## 4. Conclusions

The results obtained in the %EE, %NE, and %LC showed that method A is efficient for encapsulating the extract of chia seeds, although as shown by the zeta potential, the particles have a certain tendency to agglomerate. On the other hand, method B is less efficient than method A; however, the stability of the particles is better, and it could represent a better alternative to enlarge the lifetime of the encapsulated extract. The structural analysis performed with FTIR proved the formation of the particles and suggested the location of the extract in the particles, totally encapsulated or adsorbed on the surface. The evaluated methods allowed the obtaining of particles with a high% EE presented average sizes between ∼31 and 328 nm. The particles in which the extract was adsorbed on the surface showed antioxidant activity at 25°C. However, in the particles that only encapsulate the extract, it was observed that the acidic conditions and a temperature of 40°C allowed the release of the extract, so they presented a greater antioxidant activity. The results obtained in this work can be useful to synthesize antioxidant chia extract-loaded chitosan particles with specific characteristics according to their future applications and can serve as a basis for the encapsulation of other antioxidant extracts isolated from plant species. The particles are loaded with chia extract, which could be used as antioxidants in functional and nutraceuticals foods, in the cosmetics and pharmaceutical industries, and even in edible films for food packaging.

## Figures and Tables

**Table 1:** Encapsulation efficiency (%EE), nonencapsulated extract (%NE), loading capacity (%LC), and particle yield (%PY) of the chitosan particles loaded with the chia (*Salvia hispanica* L.) seeds extract.

Method	Particles with extract of chia (mg·mL^−1^)	%EE	%NE	%LC	%PY
A	0.0	0.0 ± 0.0^f^	0.0 ± 0.0^g^	0.0 ± 0.0^f^	38.7 ± 5.4^d^
	0.2	92.7 ± 4.5^a^	7.2 ± 4.5^f^	16.2 ± 1.5^c^	47.2 ± 4.5^c,d^
	2.0	11.8 ± 2.0^e^	88.1 ± 2.0^a^	15.1 ± 1.2^c^	51.5 ± 4.1^c^
	10.0	18.8 ± 1.7^d^	81.1 ± 1.7^b^	72.5 ± 1.8^a^	55.4 ± 1.9^c^

B	0.0	0.0 ± 0.0^f^	0.0 ± 0.0^g^	0.0 ± 0.0^f^	71.5 ± 1.6^a^
	1.0	91.6 ± 1.1^a^	8.3 ± 1.1^e^	3.2 ± 0.0^e^	69.4 ± 0.5^a^
	4.0	55.4 ± 0.9^b^	44.6 ± 0.9^d^	8.5 ± 0.2^d^	62.0 ± 1.5^b^
	15.0	52.3 ± 1.0^c^	47.6 ± 1.0^c^	26.5 ± 1.0^b^	62.3 ± 2.4^b^

The results show the means ± SD. Values with different letters as superscripts in each column are significantly different according to the Tukey test (*p* < 0.05).

**Table 2:** Zeta potential values of synthesized particles by methods A and B and chia extract-loaded particles.

Method A		Method B	
Particles with extract of chia (mg·mL^−1^)	Zeta potential (mV)	Particles with extract of chia (mg·mL^−1^)	Zeta potential (mV)
0.0	21.8 ± 0.8	0.0	26.0 ± 1.2
0.2	26.3 ± 0.3	1.0	52.4 ± 1.9
10.0	24.4 ± 0.3	15.0	39.3 ± 0.3

The results show the means ± SD.

## Data Availability

The data used to support the findings of this study are included within the article.
